# Editorial: Network Pharmacology and Traditional Medicine

**DOI:** 10.3389/fphar.2020.01194

**Published:** 2020-08-04

**Authors:** Xinxing Lai, Xin Wang, Yuanjia Hu, Shibing Su, Wenqing Li, Shao Li

**Affiliations:** ^1^MOE Key Laboratory of Bioinformatics, TCM-X Centre/Bioinformatics Division, BNRIST/Department of Automation, Tsinghua University, Beijing, China; ^2^State Key Laboratory of Quality Research in Chinese Medicine, Institute of Chinese Medical Sciences, University of Macau, Macau, Macau; ^3^Research Center for Traditional Chinese Medicine Complexity System, Shanghai University of Traditional Chinese Medicine, Shanghai, China; ^4^Key Laboratory of Carcinogenesis and Translational Research (Ministry of Education/Beijing), Department of Cancer Epidemiology, Peking University Cancer Hospital and Institute, Beijing, China

**Keywords:** network pharmacology, traditional medicine, network target, complex disease, herbal formulae

With the gradual rise of interdisciplinary subjects such as computational biology, bioinformatics, artificial intelligence, and big data science, researchers have shifted research on traditional medicine from a single and isolated mode to a multi-faceted and systematic research mode. One of the significant changes is understanding the mechanisms of drug action from the perspective of the biomolecular network. Using the “network” to regain the “whole” has brought significant changes and new challenges to medical research. Traditional medicine (TM), characterized by holistic, personalized, and multicomponent therapy, holds great potential to address a number of challenges in modern health care. By generating an unprecedented opportunity for the systematic research of TM, network pharmacology is evolving as a systematic paradigm and becoming a frontier research field of drug discovery and development. From a systematic perspective, it lays emphasis on revealing the systematic pharmacological mechanisms of drugs and further guiding the drug discovery and development, as well as clinical treatment. Network pharmacology integrates computational, experimental, and clinical investigation and creates favorable conditions for exploring the characteristics of TM and further linking to the frontiers of modern science and technology.

Network pharmacology stems from several pioneering works. The holistic theory and practice of TM play a key role in the origin and rapid development of network pharmacology. The original hypothesis referring to the biological associations between traditional Chinese medicine (TCM) syndromes, herbal formula, and molecular networks was proposed in 1999 and 2002 (Li, 1999; Li et al., 2002). On March 2006, the biomolecular networks of cold/hot syndromes were first established, and the network regulation mechanisms of hot/cold herbal formulae were illustrated experimentally (Li et al., 2007), and then a milestone article proposed a new network-based TCM research paradigm in 2007 (Li, 2007). Afterwards, the new term “network pharmacology” was introduced in Nature Biotechnology (Hopkins, 2007). Based on these pioneering works, network pharmacology was then further optimized in terms of theory, methods, database, and applications. Li proposed and continuously improved the concept and theory of “network target” (Li et al., 2011). The network target refers to a novel concept that treats the biological network underlying diseases as a therapeutic target in order to decipher systematic mechanisms of action for multi-target drugs, particularly for traditional medicine. The theory of “network target” has become the core theory of network pharmacology, exerting considerable influence in traditional medicine.

With the emerging advances in network pharmacology and traditional medicine, Frontiers in Pharmacology organized a Research Topic entitled “Network Pharmacology and Traditional Medicine” to present recent advances pertaining to network pharmacology and traditional medicine. This topic, consisted of 28 original research and two reviews, has so far attracted wide attention with over 75,000 views and more than 17,000 article downloads.

## Mechanism of Action of Herbal Formulae

A fundamental feature of TM is the use of herbal formulae as the typical treatment. Herbal formula contains hundreds of chemical compounds, which makes it complicated and challenging to understand the mechanisms of action and bioactive ingredients. The emerging network pharmacology provides a new strategy and powerful tool to uncover the biological basis underlying herbal formula. After a rigorous selection and peer review, we highlight a group of 12 original articles focus on the mechanisms of action and potential bioactive compounds of herbal formulae on a variety of complex conditions.

The first group of four papers focused on neurological or mental disorders. Among them, a paper introduced an inter-module analysis to identify an overarching view of the target profile and action mode of Huang-Lian-Jie-Du Decoction on ischemic stroke. According to the inter-module “coupling sore” analysis, a module-to-module bridge was constituted to demonstrate the “shotgun-like” pharmacological mechanism (Wang P. et al.). In addition, computational pharmacology approaches and experiments were utilized for determining the neuroprotective and anti-neuroinflammatory mechanisms underlying Tian-Ma-Gou-Teng-Yin for Alzheimer's disease (Wang T. et al.), the holistic anti-insomnia mechanism of Suan-Zao-Ren prescription by targeting multi-neurotransmitter receptors at synapse interface (Gao et al.), and key active compounds and antidepressant mechanism of Xiao-Yao-San (Yuan et al.).

Another group of four papers investigated the mechanisms underlying herbal formulae on hemopathy or infection. A paper integrated transcriptomics-based network pharmacology and metabolomics technologies to elucidate the protective effects of Qi-Jing-Sheng-Bai granule for leucopenia through accelerating cell proliferation and differentiation, regulating metabolism response, and modulating immunologic function at a system level (Tian et al.). Moreover, a computational framework of comparative network pharmacology was proposed by Chen et al. to determine the common and different mechanisms of three classic formulae for chronic liver disease, including Yinchenhao decoction, Huangqi decoction, and Yiguanjian. In addition, based on network pharmacology analysis, mechanisms and molecular targets were elucidated in terms of Yiqi Shexue formula for primary immune thrombocytopenia (Jiang et al.), and Xuebijing Injection for fungal infection-related sepsis (Shang et al.).

The last group of articles focused on gynecology, hypertensive nephropathy and aging-associated diseases. Three papers combined network analysis and *in vivo* or *in vitro* experiment to determine the mechanism of Jiawei Foshou San on inhibition of invasion and metastasis of endometriosis (Chen et al.), the protective mechanism of Li-Ru-Kang for hyperplasia of mammary gland *via* reducing damage of oxidative stress and inflammation (Wang Y. et al.), and the anti-osteoporosis effects of Erxian decoction by reducing production of TNF-α and attenuating osteoblast apoptosis (Wang N. et al.). Furthermore, based on drug perturbation of the disease biological network robustness assessment, a paper showed that Quan-Du-Zhong capsule may specifically target glomerular lesion of hypertensive nephropathy (Guo et al.).

## Herbal Active Ingredients

The development of network pharmacology has provided new perspectives in identifying and understanding the complex bioactive ingredients from numerous herbs. There are eight papers selected and published in the Research Topic to explore the molecular basis of 12 herbs.

For antitumor effect and active ingredients, two papers combined network pharmacology method and pharmacokinetics analysis, or experimental study to investigate antitumor effects and potential bioactive ingredients of *Scutellaria barbata* D. Don (Liu et al.), and the cytotoxic mechanism of krukovine for non-small cell lung cancer (Lai et al.). Another paper elucidated the antitumor effective substances and mechanism of *Phellinus igniarius* for colon cancer (Dong et al.).

Traditional medicinal herbs are commonly used for treating lipid metabolism related disorders including hyperlipidemia, hepatic steatosis, and obesity. To identify active ingredients of *Cyclocarya paliurus* (CP) leaf, a paper revealed phytochemical 14 compounds of CP by utilizing lipidomics, serum pharmacochemistry, and network pharmacology approaches (Zhai et al.). In another paper, six weight-loss herbs for obesity were investigated to identified active compounds, potential target proteins, and elucidate the pharmacological mechanism of action for obesity through a systems pharmacology framework (Zhou et al.).

Moreover, network pharmacology approaches and high performance liquid chromatography-mass spectrometry characterization were used to demonstrated that justicidin B targets the integrin α_IIb_β_3_ protein, which provided a novel perspective to understanding the mechanism on platelet aggregation (Yang et al.). Aqueous extract of the bark of *Terminalia arjuna* (TA) is widely used in the Indian subcontinent for treating cardiovascular diseases. A paper revealed the protective effects of aqueous extract of the bark TA on isoproterenol-induced cardiac hypertrophy by transcriptomic Validation (Kumar et al.).

## Effect-Enhancing and Toxicity-Reducing

Traditional herbal medicine is widely used as an adjunctive treatment for a variety of conditions, partially due to the advantages of effect-enhancing and toxicity-reducing. For toxicity-reducing, there are two papers focused on rheumatoid arthritis (RA) treatment related side effects. First, methotrexate is commonly used for RA, however, accompanied with remarkable side effects. A paper aimed to elucidate the anti-rheumatic mechanisms of Qing-Luo-Yin (QLY) and its possible interactions with methotrexate. Based on an integrating strategy coupled with network pharmacology and metabolomics techniques, it was shown that QLY notably reduced methotrexate induced side effects by eliciting antifolate resistance (Zuo et al.). Second, another paper investigated the anti-RA effect of Escin combined with glucocorticoids (GCs), which demonstrated that Escin could reduce the adverse effects of GCs (Zhang L. et al.). For effect-enhancing, Wang N. et al. revealed that Ai Du Qing significantly inhibited drug resistance in breast cancer by caveolin-1 as a key mediator, using network pharmacology and experimental validation (Wang N. et al.).

## Traditional Properties of Herbs and Formulae

One of the major challenges in terms of the modernization of herbal medicine is to uncover the scientific basis of herbal traditional properties. Four papers in this Research Topic focused on the traditional properties of Chinese medicinal herbs and formula. Among them, two papers investigated the molecular association between herbal medicine and TCM syndrome (ZHENG). First, Shen Qi Wan (SQW) is one of the most common TCM formulae in treating Kidney-Yang Deficiency syndrome (KYDS). A paper identified the potential targets of active ingredients in SQW using network pharmacology approaches, and demonstrated that SQW could regulate hypothalamic–pituitary–target gland axis disorder in KYDS rats (Zhang J. Y. et al.). Second, a constituent-target-disease network and experimental validation were conducted to identify Rk3 and 20(S)-Rg3 as the major constituents of steamed *Panax notoginseng* for blood-deficiency syndrome (Xiong et al.).

For understanding the herbal property and combinational rules, a paper selected 15 commonly used herbs attributed to spleen-meridian for chemical properties analyses to probe and clarify the theoretical basis of Chinese medicinal herbs. Base on principle component analysis of full spectrum of HPLC, NMR, and LC-MS, the findings showed that LC-MS profile was strongly correlated to the “Yin-Yang” classification criterion (Huang et al.). Furthermore, Jun-Chen-Zuo-Shi is one of the remarkable features of TCM formulae. By incorporating the pharmacokinetic and pharmacodynamics evaluation and network pharmacological analyses, a paper showed that the cooperative roles of herbs in Huo-xiang-zheng-qi prescription for functional dyspepsia conforms to the ancient compatibility rule of “Jun-Chen-Zuo-Shi” (Zhao et al.).

## Database and Methodology of Network Pharmacology

Two reviews and one article focused on network pharmacology databases and methodology for TM. First, databases and computational tools currently used for TCM research were summarized in a comprehensive review (Zhang R. et al.). The representative applications of network pharmacology for TCM research, including studies on TCM compatibility, target prediction, as well as network toxicology, were also presented in this review. Furthermore, the authors evaluated and compared the search results of several current TCM databases based on 10 famous herbs. Given the significant involvement of the gut microbiota in a broad range of diseases and the potential effect of TCM to prevent gut dysbiosis, a powerful and comprehensive database about TCM and gut microbiota is needed. Second, in the another review article contributed by Zhang W. et al., a novel approach of systems pharmacology method was proposed to identify the bioactive compounds, predict their related targets, elucidate the synergistic effects, and illustrate the molecular mechanisms of action underlying TCM (Zhang W. et al.). Third, to integrate all pieces of data and processes that allow for automatically generating mechanistic hypotheses for the known therapeutic uses of the plant, a paper integrated data linking metabolites, plants, diseases, and proteins, which provided useful systems approaches to contribute to finding a scientific rationale for traditional medicines (Olivés and Mestres).

Finally, a population-based clinical database was used to investigate the effect of Chinese herbal medicine (CHM) prescription on hip fracture, which demonstrated that CHM usage was associated with a lower risk of overall mortality, readmission, and reoperation (Cheng et al.). And the most crucial core formulae and herbs of hip fracture were identified by using association rule mining and network analysis.

## Conclusion

In conclusion, the collection of 30 articles contributed to this Research Topic illustrates the increasing attraction to network pharmacology and TM. It is important to note that the articles published in this topic cover a wide spectrum of applications of network pharmacology on TM, including the comprehensive understanding on mechanism of action of herbal formula, herbal active ingredients, molecular basis of traditional properties of herbs and herbal formulae, and mechanism underlying effect-enhancing and toxicity-reducing, as well as reviews on network pharmacology databases and tools. These articles give deep insights into the methodology and applications of network pharmacology, and also serve to present encouraging advances in this novel and promising research field. Finally, a particularly important consideration is the Four Pillars of best practice in ethnopharmacology (Frontier in Pharmacology, 2020). The evidence to evaluate the potential pharmacological effects of traditional medicine must be critically assessed in future network pharmacology studies. Furthermore, the identification of the compounds, the assessment of bioavailability of the compounds, the validation of transcriptomic and proteomic data should be conducted and reported explicitly in accordance with the Four Pillars principles.

## Author Contributions

XL and XW contributed to the concept and drafting of the manuscript. YH, SS, and WL contributed to the revision of the manuscript. SL contributed to the concept, design, and critical revision of the manuscript.

## Conflict of Interest

The authors declare that the research was conducted in the absence of any commercial or financial relationships that could be construed as a potential conflict of interest.
